# Aspects of Dietary Diversity Differ in Their Association with Atherosclerotic Cardiovascular Risk in a Racially Diverse US Adult Population

**DOI:** 10.3390/nu11051034

**Published:** 2019-05-08

**Authors:** Marie Fanelli Kuczmarski, Benjamin C. Brewer, Rita Rawal, Ryan T. Pohlig, Alan B. Zonderman, Michele K. Evans

**Affiliations:** 1 University of Delaware, Department of Behavioral Health and Nutrition, 206C McDowell Hall, Newark, DE 19716, USA; 2 University of Delaware, College of Health Sciences, STAR, Newark, DE 19716, USA; bcbrewer@udel.edu (B.C.B.); rpohlig@udel.edu (R.T.P.); 3 University of Delaware, Department of Medical Laboratory Sciences, 206C McDowell Hall, Newark, DE 19716, USA; rita@udel.edu; 4 Laboratory of Epidemiology and Population Sciences, National Institute on Aging, NIH, 251 Bayview Blvd, Baltimore, MD 21224, USA; zondermana@mail.nih.gov (A.B.Z.); evansm@grc.nia.nih.gov (M.K.E.)

**Keywords:** diet diversity, diet quality, cardiovascular disease risk, MAR, DASH

## Abstract

The study objectives were to measure dietary diversity (DD) of an urban US population and to determine if associations of 10 year atherosclerotic cardiovascular (ASCVD) risk with DD were independent of dietary quality. Participants were drawn from the Healthy Aging in Neighborhoods of Diversity across the Life Span (HANDLS) study, wave 4 (*n* = 2066, 1259 African Americans (AA), 807 Whites (W). Three DD measures were derived from 2 days of 24 h recall data collected with the USDA automated-multiple-pass-method. Count was based on consumption of at least half an equivalent of food from 21 subgroups. Evenness was calculated using Berry Index (BI) and BI-adjusted by food health values. Dissimilarity was calculated by Mahalanobis Distance. Diet quality was assessed by Mean Nutrient Adequacy (MAR) and DASH scores. Associations of DD and quality with ASCVD risk, calculated using 2013 American College of Cardiology and American Heart Association guidelines, were assessed with multivariable regression. Covariates included income, education, food security, and energy/kg weight. Count and MAR were positively associated whereas dissimilarity was negatively associated with ASCVD risk. There was no evidence that evenness contributed to cardiovascular health. The findings suggest more diversity in food attributes and diets rich in micronutrients rather than increased count support cardiovascular health.

## 1. Introduction

Diet, a collection of nutrients and other chemical substances, can have a protective role in reducing the risk of development of selected chronic conditions. Prior to the development of animal husbandry and agriculture, the universal characteristics of hominin diets can be described as minimally processed, wild plant and animal foods [1]. Industrialization, technology advancement, urbanization, globalization, and the developments in animal husbandry and agriculture transformed our food systems, reducing food biodiversity [1,2,3,4,5]. In the 1980s in the United States, dietary changes and worsening in diet quality were observed [6]. There was more reliance on processed foods, and greater use of edible oils and sugar-sweetened beverages [6].

Despite the changes in food biodiversity and availability, consuming high-quality diets can promote health. Consumption of dietary patterns based on such recommended guidelines as the Mediterranean [7,8,9,10,11,12,13] or Dietary Approaches to Stop Hypertension (DASH) [14,15,16] diet is associated with lower disease risk. In contrast, eating a Western-type diet, characterized by high consumption of red and processed meats, fried foods, refined grains/breads, added sugars, and excess alcohol is associated with high risk of disease [12,17,18].

Dietary diversity, also known as dietary variety, is globally recognized as a key component of a healthful diet. Diversity in dietary choices, provided the foods are considered healthful, would increase the potential for the provision of different nutrients and phytochemicals required for optimal health. Thus, risk for diet-related chronic conditions could be reduced. In fact, women who consumed a diversity of healthful foods had a 34% lower risk of stroke, compared to women who consumed a diet with greater quantities of less healthy foods [19].

Dietary diversity, recognized as an indicator of nutrient adequacy [20,21,22,23], has been positively associated with mean nutrient adequacy [24,25]. Dietary diversity scores have also been used to assess overall diet quality [26,27,28]. While dietary diversity appears to be a promising indicator, operationalization and measurement lack standardization and validation [29,30]. Inconsistent measures of diversity scores have contributed to the mixed results reported in the literature with respect to the association of dietary diversity and human health [31]. Some researchers have reported that dietary diversity is inversely associated with metabolic syndrome [32], obesity [33], and cardiovascular health [34,35]. Yet data from other researchers have not provided evidence to support these associations [36,37,38]. The contribution of dietary diversity to the promotion of healthful diets and optimal health is controversial and needs further exploration.

Among nutritionists the importance of dietary diversity is recognized. However, there still seems to be a lack of consensus of what dietary diversity represents. Dietary diversity scores generally evaluate variety within food groups that are usually selected from dietary guidelines [20,29]. Counts of foods or food groups have been the most frequently used method but the numbers of foods/food groups, time period, scoring, as well as the dietary collection methods varied [29]. To estimate different aspects of dietary diversity, evenness and dissimilarity have been assessed [37,39,40]. Evenness represents the distribution of foods by energy, weight or volume, sometimes adjusted by the health values of foods [30,39,40]. High evenness is desired which reflects eating a wider range of foods. Dietary dissimilarity is designed to reflect the shared or unique characteristics of food within an eating pattern. The food attributes are selected based on the health outcome of interest [37]. A high dietary dissimilarity score represents more diversity in the attributes of the foods eaten. Knowledge of these three diversity measures may better our understanding of the relationship among dietary diversity, healthful dietary patterns, and health outcomes.

The Healthy Aging in Neighborhoods of Diversity across the Life Span (HANDLS) study, a prospective longitudinal epidemiologic study, was designed with a central objective to examine the effect of socioeconomic status on health in urban-dwelling African American and White population, a population underrepresented in health research [41]. All 10 dietary patterns of this population can be characterized as Western-type. The foods contributing the highest proportion of daily energy from seven patterns (34–40%) were sandwiches, sweetened beverages, pizza, poultry (mostly fried), desserts, frozen meals, and alcoholic beverages [42]. The objectives of this study are: to assess the diversity of diets consumed by this racially and economically diverse US adult population using measures of count, evenness and dissimilarity; and to determine if these aspects of dietary diversity have associations independent of diet quality with 10-year atherosclerotic cardiovascular risk.

## 2. Methods

### 2.1. Healthy Aging in Neighborhoods of Diversity across the Life Span (HANDLS) Study Population

**Figure 1:**
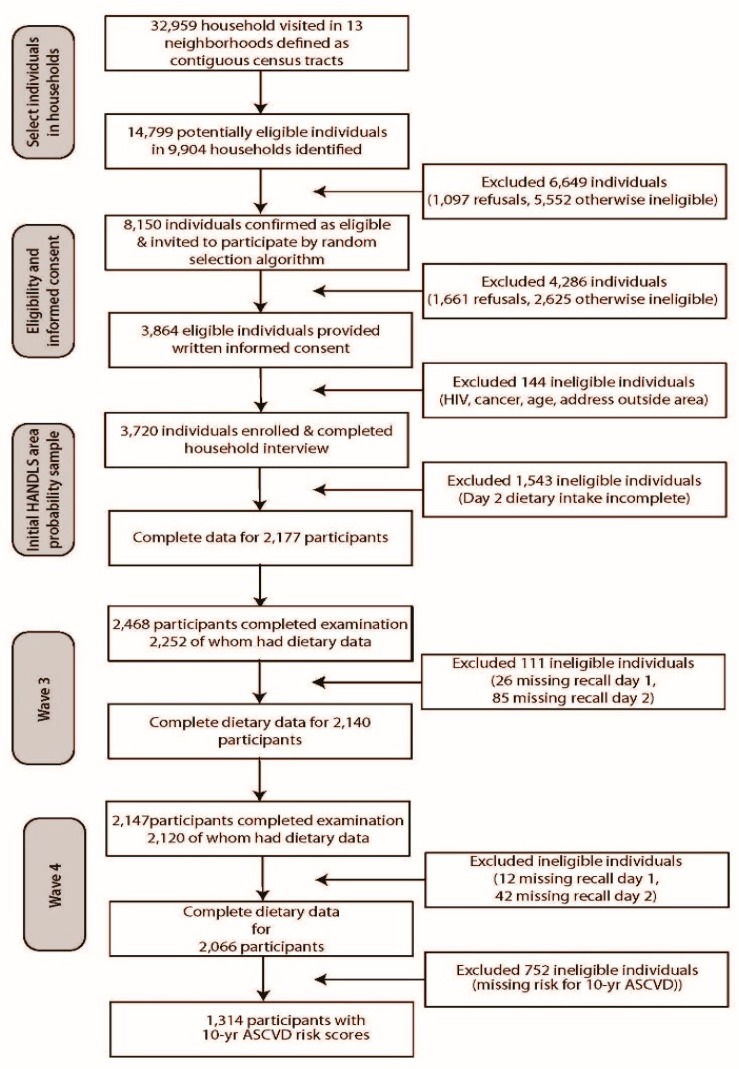
Flow diagram of household screening, participant eligibility, and response rates for the Healthy Aging in Neighborhoods of Diversity across the Life Span study. ASCVD—atherosclerotic cardiovascular disease.

The HANDLS study, initiated in 2004, is a 20-year study designed to examine the influences of race and socioeconomic status on health disparities in an urban population. The sample consisted of African American (AA) and White (W) adults, aged 30–64 years, who resided in 13 pre-determined Baltimore neighborhoods. The study design is representative of a factorial cross of four factors: age (30 to 64 years), sex (male and female), race (AA and W), and income (self-reported household income dichotomized into < and >125% of the 2004 Health and Human Services poverty guidelines [43]; hereafter termed “poverty”). The number of subjects per factorial cell is approximately equal. To date, four waves have been completed of which three were in-person examinations, namely baseline wave (2004–2009), wave 3 (2009–2013) and wave 4 (2013–2017); wave 5 began in 2017 and is ongoing. A detailed description of the study can be found elsewhere [41]. This study will use data from wave 4. As shown in Figure 1, 2066 of the 3720 participants examined in the baseline study completed both 24-h dietary recalls in wave 4 (See Figure 1.).

For each wave, the study participants completed one of two nutritional interviews and several exams and interviews in Mobile Research Vehicles (MRVs). For wave 4, the first 24-h dietary recall was completed in-person in the MRV and the second recall was done by a telephone interview. The data collected from participants in the MRV include but were not limited to demographic information, a medical examination, cognitive evaluation, physical examination, bone density, muscle strength assessments, literacy testing, and a fasting blood draw. A complete list of HANDLS variables by wave can be found elsewhere [44].

The study protocol was approved by human Institutional Review Boards (IRB) at the National Institutes of Environmental Health Sciences, National Institutes of Health, and the University of Delaware. All HANDLS participants provided written informed consent following their access to a protocol booklet in layman’s terms and a video describing all procedures. Participants were compensated monetarily.

### 2.2. Dietary Method

Both 24-h dietary recalls were collected using the United States Department of Agriculture (USDA) computerized five-step Automated Multiple-Pass Method (AMPM) [45]. The AMPM is designed to provide cues and prompts throughout the recall to capture all foods and drinks consumed the previous day [46]. These steps are described elsewhere [32]. Trained dietary interviewers conducted two 24-h dietary recalls approximately 4–10 days apart. An illustrated food model booklet, measuring cups, spoons, and a ruler were used by participants to estimate accurate quantities of foods and beverages consumed. Each 24-h dietary recall was then coded using the USDA Survey Net data processing system to match the foods with codes in the Food and Nutrient Database for Dietary Studies, 2013–14 [47]. Foods were then classified into the nine major food groups and subgroups defined in the USDA Food Patterns Equivalents Database (FPED) 2013–14 Methodology [48].

### 2.3. Diversity Measurements

The count score for a given person was characterized by the average number of foods consumed in at least one-half of a serving equivalent for 21 subgroups over that person’s two interviews. To avoid counting duplicate foods, items with same food code consumed within a 24-h period were summed prior to determining if the equivalent was eaten. Counts for the solid fats, added sugars, and alcoholic drink food groups and for the cured and organ meat subgroups were excluded from the final count since the focus of this study was diversity among food groups considered healthful; a full listing of all subgroups may be found in Table 2. The final count was calculated by the total number of subgroups consumed divided by 21; theoretical score values range from 0 to 1. Lower scores mean that fewer of the food groups were consumed, and larger scores indicate that a larger number of food groups were consumed.

Evenness scores were first estimated using the Berry Index (BI), defined as:

(1) $$1-\overset{n}{\underset{i=1}{\sum }}s_{i}^{2}$$

where $s_{i}$ is the share of food i in the total amount of energy intake and n is the total number of food items consumed [49]. Theoretical scores range from 0 to 1, with lower scores indicating that most of a participant’s daily energy came from a few food codes and higher scores indicating that a large number of food codes contributed equally to a participant’s daily energy consumption.

Evenness scores were then estimated by incorporating health factors [HF]; health factors were assigned to each food subgroup based on the Dietary Guidelines for Americans, 2015–2020 [50], and are listed in Supplementary Table S1. A weighted average based on the number of equivalents of each food subgroup in a given food code was used to determine the health factor for that food code. These health factors were then used to adjust the value of the Berry Index using the following formula from Drescher and colleagues. [39]

(2) $$HFBI=\frac{\left(1-\sum_{i=1}^{n}s_{i}^{2}\right)\left(\sum_{i=1}^{n}hf_{i}\times s_{i}\right)}{\underset{i}{\max}\left(hf_{i}\right)}=\frac{\left(BI\right)\;\times \;\left(HV\right)}{0.18009}$$

Division by the maximum possible health factor value ensures that the theoretical scores range between 0 and 1, with higher scores indicating not only equal energy contribution from many food codes but also that the foods consumed were considered to be healthy. Scores decrease as the overall healthfulness of the foods decrease and/or the daily energy contribution shifts to a relatively small number of food codes.

Dissimilarity scores were found by finding the average distance between all pairs of foods consumed by an individual across 10 attributes relevant to cardiovascular health, reflecting selected nutrient composition, source, or processing of a food. The attributes included: animal protein, plant protein, whole grains, refined grains, eicosapentaenoic acid (EPA)/docosahexaenoic acid (DHA), dietary fiber, sodium, alcohol, solid fats, and oils. Each attribute was scored as either a 0 or 1 for every food code. Fiber, sodium, and EPA/DHA attribute scores were calculated based on the gram content of that attribute within a given food code. The other seven attributes were determined based on food subgroups with nonzero equivalents for a given food code; equivalents were taken from the USDA’s FPED database. See Supplementary Table S2 for definitions and cutoff points for each attribute.

Distance was calculated using Mahalanobis Distance (MD), a way to get the standardized distance between two points in multivariate space adjusting for the correlation among the variates [51]. It is defined here as:

(3) $$\sqrt{{\left(x_{i}-y_{i}\right)}^{T}\sum^{-1}\left(x_{i}-y_{i}\right)},$$

where $x_{i}$ is the vector of attribute values for food x, $y_{i}$ is the vector of attribute values for food y, and ∑ is the variance-covariance matrix among the attributes.

Since all of the attributes were dichotomous, the covariance matrix used was calculated using a method proposed by Schweizer [52]. This method assumes that the observed dichotomous values are just indicators of a continuous normally distributed underlying latent construct. MD was chosen for this study to account for the fact that attributes were correlated. Most distances ranged from 0 to 1.5, with larger scores indicating that a person’s diet consisted of foods with a greater variety of attribute values. While theoretical values of MD can range from 0 to ∞, larger values (≥3) are extremely unlikely. In this context, this is because MD essentially gives the number of standard deviations that a given food is away from the “attribute average,” and most foods will fall within one or two standard deviations from this average.

### 2.4. Diet Quality Measures

Mean Adequacy Ratio (MAR) scores were based on Nutrient Adequacy Ratios (NAR) for 17 micronutrients [28,42]. NAR score is the participant’s daily intake of a nutrient divided by the Recommended Dietary Allowance (RDA) for that nutrient. The RDA selected matched the age and sex of the participant [53]. If the participant was a smoker, his/her RDA for vitamin C was adjusted upward by an additional 35 mg [54]. The calculation of the NAR and MAR scores are described in detail in a previous publication [42]. The maximum NAR and MAR scores were 100. MAR scores were based on food intakes only and are representative of nutrient-based diet quality.

Diet quality was also measured by the score for DASH diet adherence for each participant using the formula reported by Mellen and colleagues [55]. The maximum DASH score was 9; individuals were considered DASH adherent if his/her score met half or more of the DASH targets (DASH score ≥4.5) [55].

### 2.5. Demographic and Health-Related Measures

Demographic characteristics measured included age (years), sex, race, poverty status, education, and smoking status. Race was self-reported as AA or W. Self-income was categorized as either <125% or >125% poverty [43]. Education was categorized as <12^th^ grade education or ≥12^th^ grade education/general equivalency diploma. Food insecurity was defined as a positive response to one question, “In the past 12 months, did you ever eat less than you felt you should because there was not enough money to buy food?”

A calibrated Med-weigh, model 2500 digital scale was used to measure weight. A height meter (Novel Products, Inc) was used to measure height with the participant standing so heels and back were against the meter. BMI was calculated as the ratio of weight (kg) to height (m) squared and analyzed as a continuous measure.

Ten-year risk was defined as the risk of developing a first Atherosclerotic Cardiovascular Disease (ASCVD) event over a 10-year period among HANDLS study participants free from ASCVD at wave 4. The ASCVD event was defined as nonfatal myocardial infarction or coronary heart disease death, or fatal or nonfatal stroke. Risk was calculated using the pooled cohort equations published by the Expert Work Group of the American College of Cardiology and American Heart Association. These equations only apply to persons aged 40–79 years [56]. Age, race, sex, total and HDL-cholesterol, systolic blood pressure, diabetes, treatment for hypertension, and current smoking status were variables included in these equations. Therefore, these variables were not in the regression models.

### 2.6. Statistical Analyses

Means and standard errors for continuous variables and proportion of participants for relevant categorical variables were calculated. The correlations between each of the three dietary diversity scores and each of the two diet quality measures were calculated. A test statistic based on Pearson’s product moment correlation coefficient (which follows a t-distribution with n−2 degrees of freedom) was used to determine if the correlation was significantly different from 0. This test was performed in R using the *cor.test* function [57].

Analysis of variance (ANOVA) was used to compare age, BMI, and diet first across the levels of race and then across the levels of income. For categorical data, chi-square tests were used. The Wilcoxon Rank-Sum Test was used to compare the means of the dietary diversity scores (count, evenness (BI), health-value-adjusted evenness (HFBI), and dissimilarity) first across the levels of race and then across the levels of income; this test is approximately as powerful as the t-test in situations where the t-test is appropriate; however, unlike the t-test, it is robust in situations where the data deviate from normality [58,59]. This means that no parametric assumptions are required of these data in order to determine significance.

Two regression models were used for 10-year risk for ASCVD. The first model included MAR and the second model used DASH for indices of diet quality. These models tested the associations of diet diversity, count score, health-value-adjusted evenness score, and dissimilarity, education (<12^th^ grade or ≥12^th^ grade), energy consumed per kilogram of body weight, food insecurity status, and income status (<125% of poverty or >125% of poverty) with ASCVD risk. The assumptions for the methods were assessed using residual analysis for normality, outliers, and homoscedasticity. Multicollinearity was assessed using variance inflation factors [60].

Statistical significance was established using *p* < 0.05. All statistical analyses were performed with either IBM SPSS Statistics for Windows v25 (summary statistics) (2017; IBM Corporation, Armonk, NY, USA) or R v3.5.2 (multivariable models) (2018; R Foundation for Statistical Computing, Vienna).

## 3. Results

### 3.1. Population Characteristics

The mean age of the wave 4 study population who completed two dietary recalls (*n* = 2066) was approximately 57 years (Table 1). Comparison of race and income groups revealed no differences in proportion of those with less than a high school (<12^th^ grade) education. The percentage of women and those who reported food insecurity were significantly higher in the <125% poverty group compared to those with incomes >125% poverty. The mean (± SE) 10-year ASCVD risk was 56.2 ± 0.2% and did not differ by race. However, persons with higher incomes had significantly greater risk than those with income <125% poverty (Table 1). The mean body mass index of the group was 31, indicating obesity, and did not differ between racial or income groups (data not shown).

### 3.2. Dietary Characteristics

The mean (± SE) energy intake of the HANDLS study population was approximately 1965 ± 18 kcal and did not differ by race or income (Table 1). The mean (± SE) MAR and DASH scores were 73.9 ± 0.3 out of 100 and 2.04 ± 0.03 out of 9, respectively. Both diet quality measures were significantly higher in Whites compared to AAs and in the higher income group (>125% poverty) compared to the <125% poverty group. There was no difference in adherence to the DASH diet and less than 6% of the population was adherent (Table 1). There was less than a 2% change in diet quality scores over the three study waves (unpublished data).

The mean equivalents for major food groups and their subgroups are provided in Table 2. The subgroups with the greatest mean equivalents were other fruits, other vegetables, refined grains, poultry, and cheese. The percentage of energy contributed by the five major food groups is provided in Table 3. Grains provided the highest percent (~32%), followed by protein foods, vegetables, dairy, and lastly, fruits (~4%). Counts for the major food groups and oils categorized by race and income are also provided in Table 3. The count for total protein food group was significantly higher for AA compared to W, while W had the higher count for dairy group. Except for oils, the count of each of the major groups was greater for persons >125% poverty compared to those <125% poverty.

Mean (± SE) diversity scores were as follows: 0.445 (± 0.100) for count, 0.808 (± 0.079) for evenness-BI, 0.128 (±0.051) for evenness-HFBI, and 0.802 (± 0.078) for dissimilarity. As shown in Table 1, mean count was significantly higher for persons with income > 125% poverty compared to those with income <125% poverty but did not differ by race. The mean evenness score based on the Berry Index also differed by income but not race. When the Berry Index was adjusted for the health value of foods, the score decreased and did not differ by either race or income. Whites had a significantly greater dissimilarity score compared to AAs (Table 1).

All aspects of diet diversity were weakly correlated with each other; these correlations were significantly different from 0 (Supplementary Table S3). The correlation of count with evenness-HFBI was 0.167 (*p* < 0.001) and with dissimilarity was 0.209 (*p* < 0.001). The correlation between evenness- HFBI and dissimilarity was −0.043 (*p* = 0.049). The magnitude of all correlations between diet quality measures and diversity aspects were weak to moderate (< 0.270) except for the correlation of MAR with count which was 0.529. These correlations were also all significantly different from 0 except for the correlation between the count score and the DASH score (*r* = −0.003, *p* = 0.898). Several food attributes were also moderately correlated (Supplementary Table S4); some examples involve animal proteins and solid fats (*r* = 0.458), moderate/high sodium and refined grains (*r* = 0.317), and moderate/high sodium and solid fats (*r* = 0.338).

All three aspects of diet diversity, namely count, evenness-HFBI, and dissimilarity, and diet quality characterized by either MAR or DASH along with education, income, food security and energy per kg body weight were included in the regression models. Regardless of which diet quality index was included, education, income, and evenness were not significant predictors of 10-year ASCVD risk (all *p* > 0.05). For both indices count was significantly positively associated with risk, and dissimilarity was significantly negatively associated with risk (all *p* < 0.03). Being food secure was associated with higher 10-year ASCVD risk in both models (Table 4 and Table 5). In the model which included MAR as the diet quality measure, energy per kg body weight was not significant. Higher diet quality characterized by MAR was associated with lower ASCVD risk (Table 4). These results were reversed when DASH was included in the model. Diet quality assessed by DASH score was not significant but consuming less energy per kg body weight was associated with higher 10-year ASCVD risk (Table 5).

## 4. Discussions

In this study, we found that some aspects of dietary diversity independent of quality were associated with 10-year ASCVD risk. The study is novel in that it included two measures of diet quality in addition to three aspects dietary diversity. The findings provide evidence that count and dissimilarity but not evenness were associated with ASCVD risk. MAR, a dietary quality measure based on the micronutrient intake in relation to recommended intakes, but not DASH, a dietary quality measure based on a healthy eating pattern, was also associated with lower disease risk. The lack of association between DASH score and 10-year ASCVD risk may reflect the lack of variance within DASH scores given <6% of the population were adherent to the DASH eating plan. However, neither MAR nor DASH scores reveal “real” nutritional status.

The study findings provide evidence for the benefit of dissimilarity for cardiovascular health. Greater dissimilarity among foods was associated with lower 10-year ASCVD risk. Dissimilarity in this study captured diversity in types of foods consumed based on shared or unique attributes relevant to cardiovascular health. This finding is different from that reported by Otto and colleagues [37]. They reported a positive association between dissimilarity and gain in abdominal obesity, suggesting that eating everything in moderation did not confer better metabolic health. An explanation of the inconsistency in findings may be attributed to the difference in the dissimilarity formula, the number of food attributes selected as well as the definition of attributes and outcome variable chosen. While Otto and colleagues oftentimes used attribute tertiles of 100 g of food consumption, this study used nutrient composition per serving to develop food attribute criteria [37]. To be able to compare findings across studies, there is a need to develop a standardized method for measuring dissimilarity.

In the HANDLS cohort, the health factor-adjusted BI evenness scores were lower than the unadjusted BI scores. This finding was consistent with those reported by Drescher and colleagues [39] despite the use of different health factors. When the distribution between food groups favors healthful food groups, the evenness score will increase. The observed drop in the evenness score indicated that change in distribution favored unhealthful food items. Unlike the findings of others [39,61,62,63], in this study evenness was not associated with a health outcome.

Counts reflect the number of different foods consumed. It would be anticipated that greater variety would lessen disease risk provided the foods have a nutrient profile which optimizes cardiovascular health. However, this study found that increasing count was associated with higher ASCVD risk. An explanation for this association is that the food consumed does not reflect the lowest energy, nutrient-richest options. Indeed based on the dietary data from two previous waves of the HANDLS study, the dietary patterns of the study population have been characterized as Western-type [42,64]. This finding supports the concept proposed by Otto and colleagues that everything in moderation does not lead to better health and others who have reported higher counts were associated with weight gain and obesity [38,65].

The importance of food security is recognized by the Food and Agricultural Organization of the United Nations in ending hunger (Sustainable Development Goal 2) and ensuring healthy lives and promoting well-being (Sustainable Development Goal 3) [66]. Food insecurity has been associated with energy-dense and nutrient-poor diets and suboptimal nutrition [67]. This study found that being food secure was associated with higher ASCVD risk. This finding was not totally unexpected for the study sample. Individuals who were food secure had incomes above poverty with maximum household incomes of approximately $70,000. Two out of three adults in the above poverty group, compared to one in three persons in the below poverty group, reported alcoholic beverage consumption on recall days. The mean daily alcohol consumption was ~34 g for both income groups. It is recognized that the mechanisms by which alcohol consumption may exert cardiovascular effects are complex. The association between alcohol consumption and total cardiovascular disease risk comprises several distinct and opposite dose-response curves. Although there are no clear risk thresholds below which lower alcohol consumption stops being associated with lower cardiovascular disease risk, among current drinkers, the threshold for lowest risk of all-cause mortality has been reported to be about 100 g per week [68]. Based on daily intakes of 34 g, this weekly threshold would be exceeded by HANDLS study participants.

There are many strengths of this study. Dietary diversity measures included count, evenness and dissimilarity measures and were based on two 24-h dietary recalls which provide more detail about foods consumed compared to the food frequency methods. Additionally, the use of the Mahalanobis Distance for dietary dissimilarity rather than the Jacquard index accounted the correlation between attributes, eliminating double counting of attributes. Count and evenness measures were focused on healthful foods. As with all research, there are limitations. The dietary collection method used the USDA AMPM which has been shown to reduce bias in the collection of energy intakes, and to provide accurate estimates of sodium intake [45,69]. Nevertheless, inherent errors are associated with the 24-h recall and social desirability can affect reporting of intake behaviors [70]. Although the HANDLS study is longitudinal, the results are only based on wave 4. Therefore, no casual inferences can be made. Future research with longitudinal data from three study waves will be able to address if dietary diversity measures are predictive of risk for 10-year ASCVD.

## 5. Conclusions

Despite the consumption of a Western-type diet, the study findings suggest that more diversity in food attributes and diets rich in micronutrients support cardiovascular health. Even among persons with poor food choices it seems it is still possible that small dietary differences may reduce risk for cardiovascular disease. Increased count of items comprising the Western-type diet were associated with increased risk of 10-year ASCVD. If more individuals in this urban sample could achieve food security they may consume a diet of better quality which might result in lower 10-year ASCVD risk. For those who are food secure, a better understanding of the contribution their food choices to disease risk may result in change to a more healthful diet.

## Figures and Tables

**Table 1:** Sociodemographic and dietary characteristics of HANDLS study population (*n* = 2066).

Characteristic	Race			Income		
	AA *n* = 1259	W *n* = 807	*p*	<125% Poverty *n* = 842	>125% Poverty *n* = 1224	*p*
Age, year	56.7 ± 0.3	56.6 ± 0.3	0.875	56.0 ± 0.3	57.1 ± 0.3	**0.008**
Sex, % female	59.1	58.9	0.916	63.4	56.0	**0.001**
Education, % <high school	30.9	32.7	0.386	32.3	31.1	0.572
Food Insecurity, % insecure^1^	27.1 *n* = 1125	24.2 *n* = 744	0.159	31.3 *n* = 739	22.5 *n* = 1130	**<0.001**
Energy, kcal	1948 ± 23	1992 ± 29	0.237	1945 ± 29	1979 ± 23	0.350
Diversity: Count	0.4446	0.4445	0.934	0.4287	0.4558	**<0.001**
Diversity: Evenness (HFBI)	0.1284	0.1285	0.700	0.1277	0.1290	0.295
Diversity: Evenness (BI)	0.8078	0.8093	0.824	0.8034	0.8119	**0.007**
Diversity: Dissimilarity^2^	0.8063	0.7944	**<0.001**	0.8056	0.7990	0.056
Mean Nutrient Adequacy	73.1 ± 0.4	75.2 ± 0.5	**0.001**	72.6 ± 0.5	74.8 ± 0.4	**0.001**
DASH score	1.90 ± 0.03	2.25 ± 0.04	**<0.001**	1.94 ± 0.04	2.11 ± 0.04	**0.001**
DASH, % adherent^3^	4.1	5.8	0.064	3.8	5.4	0.094
ASCVD risk, %	56.2 ± 0.3 *n* = 778	56.3 ± 0.3 *n* = 536	0.864	55.5 ± 0.4 *n* = 509	56.7 ± 0.3 *n* = 805	**0.012**

Abbreviations: AA—African Americans, BI—Berry Index, DASH—Dietary Approaches to Stop Hypertension, HANDLS—Healthy Aging in Neighborhoods of Diversity across the Life Span, HFBI—Health Factor adjusted Berry Index, W—Whites. ^1^ Defined by affirmative response to question, ‘Did you eat less because of insufficient money for food in the past month?’. ^2^Defined by Mahalanobis Distance [51]. ^3^ Calculated formula reported by Mellen and colleagues [55]. Bolded font was used to emphasize *p*-values significant at <0.05.

**Table 2:** Mean daily equivalents consumed for each food group by HANDLS study population.

Food Group	Mean Equivalents	Food Group	Mean Equivalents
Total Fruit	0.126 cup	Total Protein foods	2.129 oz
Citrus, melons, berries	0.032 cup	Total Meat, poultry, fish^1^	1.488 oz
Other fruits	0.065 cup	Meat	0.181 oz
Juices	0.030 cup	Cured meat^1^	0.466 oz
Total vegetables	0.767 cup	Organ meat^1^	0.006 oz
Dark green	0.125 cup	Poultry	0.757 oz
Total red and orange	0.212 cup	Seafood high in n-3 fatty acids	0.014 oz
Total starchy	0.051 cup	Seafood low in n-3 fatty acids	0.064 oz
Other vegetables	0.368 cup	Eggs	0.065 oz
Legumes	0.010 cup	Soy products	0.012 oz
Total grains	1.360 oz	Nuts and seeds	0.564 oz
Whole grains	0.238 oz		
Refined grains	1.122 oz	Oils	20.120 g
Total Dairy	2.990 cup		
Milk	0.138 cup	Solid fats^1^	45.571 g
Yogurt	0.001 cup	Sugars + Beverages^1,2^	26.648 tsp
Cheese	2.845 cup	Alcoholic drinks^1^	0.009 drinks

Abbreviations: HANDLS—Healthy Aging in Neighborhoods of Diversity across the Life Span. ^1^ Excluded from count score. ^2^ Includes non-alcoholic beverages other than water.

**Table 3:** Summary of counts by race and income for HANDLS study population (*n* = 2066).

Food Group	Energy, % Total^1^	Race			Income		
		AA	W	*p*	<125% Poverty	>125% Poverty	*p*
Total grains	32.16	0.7077	0.7088	0.8392	0.6900	0.7206	**<0.0001**
Total protein foods^2^	27.33	0.3344	0.3197	**<0.0001**	0.3207	0.3342	**0.0015**
Total vegetables	8.80	0.4558	0.4539	0.7581	0.4305	0.472	**<0.0001**
Total dairy	8.31	0.4617	0.5118	**<0.0001**	0.4677	0.4906	**0.0035**
Total fruit	3.65	0.3119	0.3036	0.1958	0.2819	0.3271	**<0.0001**
Oils	2.04	0.9805	0.9734	0.1269	0.9762	0.9788	0.5909

Abbreviations: AA—African Americans, HANDLS—Healthy Aging in Neighborhoods of Diversity across the Life Span, W—Whites. ^1^ Percent of energy contributed by remaining food groups was 1.01% solid fats, 13.34% for sugars and sweetened beverages, 3.35% for alcoholic beverages. ^2^ Excluded processed and organ meats. *p*-value <0.05 are in bold.

**Table 4:** Association of 10-year ASCVD risk with diet diversity, diet quality as Mean Nutrient Adequacy and selected sociodemographic covariates: Multivariable Regression Model.

Covariate	Estimate	SE	*p*
Education (<high school vs. ≥high school)	−0.458	0.467	0.327
Energy per kg body weight	−0.033	0.023	0.148
Food security (insecure vs. secure) ^1^	2.615	0.53	**<0.001**
Income (>125% poverty vs. <125% poverty)	0.848	0.468	0.07
Count	11.746	2.666	**<0.001**
Evenness—Health Factor-adjusted Berry Index	9.055	4.736	0.056
Dissimilarity^2^	−6.301	3.051	**0.039**
Mean Adequacy Ratio	−0.127	0.022	**<0.001**

Abbreviation: ASCVD—Atherosclerotic Cardiovascular Disease. ^1^ Defined by affirmative response to question, ‘Did you eat less because of insufficient money for food in the past month?’. ^2^ Defined by Mahalanobis Distance [51]. Bolded font was used to emphasize *p*-values significant at <0.05.

**Table 5:** Association of 10-year ASCVD risk with diet diversity, diet quality as DASH score and selected sociodemographic covariates: Multivariable Regression Model.

Covariate	Estimate	SE	*p*
Education (<high school vs. ≥high school)	−0.536	0.472	0.256
Energy per kg body weight	−0.101	0.02	**<0.001**
Food security (insecure vs. secure) ^1^	2.442	0.535	**<0.001**
Income (>125% poverty vs. <125% poverty)	0.748	0.474	0.115
Count	5.289	2.427	**0.030**
Evenness- Health Factor-adjusted Berry Index	8.146	4.861	0.094
Dissimilarity ^2^	−8.875	3.25	**0.006**
DASH score ^3^	−0.395	0.216	0.067

Abbreviations: ASCVD—Atherosclerotic Cardiovascular Disease, BI—Berry Index, DASH—Dietary Approaches to Stop Hypertension. ^1^ Defined by affirmative response to question, ‘Did you eat less because of insufficient money for food in the past month?’. ^2^ Defined by Mahalanobis Distance [51]. ^3^ Calculated formula reported by Mellen and colleagues [55]. Bolded font was used to emphasize *p*-values significant at <0.05.

## Supplementary Materials

The following are available online at https://www.mdpi.com/2072-6643/11/5/1034/s1, **Table S1**: Health Values of Foods, **Table S2**: Definitions of Food Attributes, **Table S3**: Correlation matrix of dietary diversity and quality measures, **Table S4**: Correlation matrix of food attributes.
