# Correction: Fanelli Kuczmarski, M.; et al. Aspects of Dietary Diversity Differ in Their Association with Atherosclerotic Cardiovascular Risk in a Racially Diverse US Adult Population. *Nutrients* 2019, *11*, 1034

**DOI:** 10.3390/nu11112609

**Published:** 2019-10-31

**Authors:** Marie Fanelli Kuczmarski, Benjamin C. Brewer, Rita Rawal, Ryan T. Pohlig, Alan B. Zonderman, Michele K. Evans

**Affiliations:** 1 University of Delaware, Department of Behavioral Health and Nutrition, 206C McDowell Hall, Newark, DE 19716, USA; 2 University of Delaware, College of Health Sciences, STAR, Newark, DE 19716, USArpohlig@udel.edu (R.T.P.); 3 University of Delaware, Department of Medical Laboratory Sciences, 206C McDowell Hall, Newark, DE 19716, USA; rita@udel.edu; 4 Laboratory of Epidemiology and Population Sciences, National Institute on Aging, NIH, 251 Bayview Blvd, Baltimore, MD 21224, USA; zondermana@mail.nih.gov (A.B.Z.); evansm@grc.nia.nih.gov (M.K.E.)

The authors wish to make a correction to Table 2 in the published version of their paper [1].

A programming error was found when estimating mean equivalents from other HANDLS study waves. The mean equivalents were calculated based on only the last intake of a food and not the daily intake. A corrected version of Table 2 is shown below. 

This change does not affect the dietary diversity scores, the overall results of the regression analyses or scientific conclusions since mean equivalents were not used in score calculations. However, one edit to the second sentence in the second paragraph of *3.2. Dietary Characteristics* is needed. The mean equivalents of starchy vegetables exceeded other vegetables. The sentence should now read, “The subgroups with the greatest mean equivalents were other fruits, total starchy vegetables, refined grains, poultry and cheese.”

The authors would like to apologize for any inconvenience caused to readers by these changes.

## Figures and Tables

**Table 2:** Mean daily equivalents (± standard errors) consumed for each food group by HANDLS study population.

Food Group	Mean ± SE Equivalents	Food Group	Mean ± SE Equivalents
Total Fruit	0.745 ± 0.022 cups	Total protein foods	6.539 ± 0.084 oz
Citrus, melons, berries	0.116 ± 0.008 cups	Total meat, poultry, fish ^1^	5.146 ± 0.073 oz
Other fruits	0.338 ± 0.013 cups	Meat	1.202 ± 0.040 oz
Juices	0.291 ± 0.014 cups	Cured meat ^1^	1.243 ± 0.033 oz
Total vegetables	1.329 ± 0.021 cups	Organ meat ^1^	0.032 ± 0.007 oz
Dark green	0.166 ± 0.008 cups	Poultry	1.686 ± 0.045 oz
Total red and orange	0.280 ± 0.007 cups	Seafood high in n-3 fatty acids	0.236 ± 0.019 oz
Total starchy	0.470 ± 0.012 cups	Seafood low in n-3 fatty acids	0.747 ± 0.036 oz
Other vegetables	0.361 ± 0.009 cups	Eggs	0.668 ± 0.016 oz
Legumes	0.052 ± 0.004 cups	Soy products	0.035 ± 0.004 oz
Total grains	5.439 ± 0.064 oz	Nuts and seeds	0.482 ± 0.033 oz
Whole grains	0.668 ± 0.021 oz	Legumes	0.208 ± 0.016 oz
Refined grains	4.771 ± 0.062 oz		
Total Dairy	1.154 ± 0.023 cups	Oils	25.657 ± 0.384 g
Milk	0.494 ± 0.015 cups	Solid fats ^1^	34.672 ± 0.482 g
Yogurt	0.039 ± 0.004 cups	Sugars + beverages ^1,2^	19.766 ± 0.328 tsp
Cheese	0.621 ± 0.015 cups	Alcoholic drinks ^1^	0.509 ± 0.035 drinks

Abbreviations: HANDLS—Healthy Aging in Neighborhoods of Diversity across the Life Span, SE—standard error. ^1^ Excluded from count score. ^2^ Includes non-alcoholic beverages other than water.

## References

[1] Fanelli Kuczmarski M., Brewer B.C., Rawal R., Pohlig R.T., Zonderman A.B., Evans M.K. (2019). Aspects of Dietary Diversity Differ in Their Association with Atherosclerotic Cardiovascular Risk in a Racially Diverse US Adult Population. Nutrients.

