# Supplementary material for: Aspects of Dietary Diversity Differ in Their Association with Atherosclerotic Cardiovascular Risk in a Racially Diverse US Adult Population
Source: Nutrients. 2019 May 8;11(5):1034. doi: 10.3390/nu11051034 (PMC6566273; doi:10.3390/nu11051034)
Supplement: Supplementary file 1 [file nutrients-11-01034-s001.pdf]

Table S1. Health values of foods.

Food group	equivalents per week[1]	%	Health value
Total Fruit[2]	14	10.8	
Citrus, melons, berries	7	50.0	0.108 × 0.5 = 0.054
Other fruits	4	28.6	0.108 × 0.286 = 0.031
Juices[3]	3	21.4	0.108 × 0.214 = 0.023
Total vegetables	17.5	13.5	
Dark green	1.5	8.6	0.135 × 0.086 = 0.012
Total red and orange	5.5	31.4	0.135 × 0.314 = 0.042
Total starchy	5	28.6	0.135 × 0.286 = 0.039
Other vegetables	4	22.9	0.135 × 0.229 = 0.031
Legumes	1.5	8.6	0.135 × 0.086 = 0.012
Total grains	42	32.4	
Whole grains	21	50	0.324 × 0.50 = 0.162
Refined grains	21	50	0.324 × 0.50 = 0.162
Total Protein foods	39	27.0	
Meat, poultry, eggs[4]	26	66.7	0.270 × 0.667 = 0.180
Seafood high & low in n-3 fatty acids	8	20.5	0.270 × 0.205 = 0.055
Soy products, nuts, and seeds	5	12.8	0.270 × 0.128 = 0.035
Total Dairy[5]	21	16.2	
Milk	1	33.3	0.162 × 0.333 = 0.054
Yogurt	1	33.3	0.162 × 0.333 = 0.054
Cheese	1	33.3	0.162 × 0.333 = 0.054
Oils	0.125 C	0.1	0.1
Equivalents/ week	129.625	100	

[1] Based on 2000 kcal diet from 2015–2020 Dietary Guidelines for Americans [1]. [2] Values based on 2015–2020 Dietary Guidelines for Americans which states at least half of the recommended amount of fruits should come from whole fruits [2]. Since citrus was one of the Basic Seven food groups in 1940s, this group was weighted more heavily. The value of 7 suggests one equivalent serving of citrus per day. [3] Reflects consumption of adults from scientific report 2015–2020 Dietary Guidelines for Americans [2]. [4] Excluded cured and organ meats. [5] There is no recommendation for dairy subgroups in the Dietary Guidelines for Americans, 2015–2020. Equivalents were distributed equally across the subgroups based on the following rational. The Scientific Advisory Report of dietary guidelines for 2015–2020 noted that the U.S. population consumes the recommended 3 cup equivalents per day as 53 percent fluid milk, 45 percent cheese, and 2 percent as yogurt [3]. These data support approximately equally consumption of milk and cheese. Yogurt consumption is low but research that documents the benefits of yogurt with respect to heart health [3].

Table S2. Definitions of food attributes.

Attribute	Categories	Criterion to Assess
Animal protein source	Yes—Coded as 1[1]<br>No—Coded as 0	USDA Food Patterns Equivalent Database food subgroups[2]<br>Protein Foods, Meat<br>Protein Foods, Poultry<br>Protein Foods, Seafood High in EPA/DHA<br>Protein Foods, Seafood Low in EPA/DHA<br>Protein Foods, Eggs<br>Dairy, Milk<br>Dairy, Yogurt<br>Dairy, Cheese
Plant protein source	Yes—Coded as 1[1]<br>No—Coded as 0	USDA Food Patterns Equivalent Database food subgroups[2]<br>Soy Products<br>Nuts and Seeds
Food processing: Whole grains	Yes—Coded as 1<br>No—Coded as 0	USDA Food Patterns Equivalent Database food subgroups[2]<br>Whole Grains
Food processing: Refined grains	Yes—Coded as 1<br>No—Coded as 0	USDA Food Patterns Equivalent Database food subgroups[2]<br>Refined Grains
Fiber	Initially coded as:<br>High—Coded as 1;<br>Moderate—Coded as 2; Low—Coded as 3; No Fiber: Coded as 0;<br>For analysis coded as: High to moderate fiber—Coded as 1; Low to No fiber<br>—Coded as 0	Comparison of fiber content to the amount in serving size.<br>FDA defines serving size as the amount of food typically consumed in one sitting for that food and they are determined using Reference Amounts Customarily Consumed (RACC) and procedures described in 21 CFR 101.12(b) and 21 CFR 101.9(b) respectively.[3]<br>Using FDA labeling criterion per serving[3]<br>High fiber food is defined as ≥5 g of fiber;<br>Moderate fiber food is defined as 1.26–4.9 g of fiber;<br>Low fiber food is defined as ≤1.25 g of fiber
Sodium	Initially coded as:<br>High—Coded as 1<br>Moderate—Coded as 2<br>Very Low—Coded as 3<br>No sodium—Coded as 0<br>For analysis coded as:<br>High to moderate sodium<br>—Coded as 1<br>Very Low to No sodium<br>—Coded as 0	Comparison of sodium content to the amount in serving size.<br>FDA defines serving size as the amount of food typically consumed in one sitting for that food and they are determined using Reference Amounts Customarily Consumed (RACC) and procedures described in 21 CFR 101.12(b) and 21 CFR 101.9(b) respectively.[3]<br>Using FDA labeling criterion per serving[3],<br>High sodium food is defined as >140 mg of sodium;<br>Moderate sodium food is defined as 36–140 mg;<br>Very low sodium food is defined as ≤ 35 mg of sodium
Alcohol	Yes—Coded as 1<br>No—Coded as 0	Using USDA Food Patterns Equivalent Database food groups[2], only foods listed under Alcoholic Drinks Components to be used to categorize foods as having alcohol or not.
Eicosapentaenoic acid (EPA)<br>Docosahexaenoic acid (DHA)	Initially coded as: High—To be coded as 1; Low—To be coded as 2; No EPA + DHA be coded as 0<br>For analysis coded as: High EPA/DHA—Coded as 1; Low to No EPA/DHA—Coded as 0	Comparison of total EPA+DHA content to the amount in serving size.<br>High EPA + DHA food is defined as having ≥ 0.90 g of EPA + DHA per serving4<br>Low EPA + DHA food is defined as having 0.01–0.89 g of EPA + DHA per serving[4]
Oils	Yes—Coded as 1<br>No—Coded as 0	USDA Food Patterns Equivalent Database food groups[2]<br>Oils
Solid fats	Yes—Coded as 1<br>No—Coded as 0	USDA Food Patterns Equivalent Database food groups[2]<br>Solid fats

[1]If a given food code had a nonzero equivalent value in *at least* one of these subgroups, the corresponding attribute was assigned a value of "1" for that food code. If all qualifying subgroups had zero equivalents, the corresponding attribute for that food code was assigned a value of "0". [2]Food Patterns Equivalents Database 2013–14: Methodology and User Guide [4]. [3]Food Labeling: Serving sizes of Foods That Can Reasonably Be Consumed at One Eating Occasion; Dual-Column Labeling; Updating, Modifying, and Establishing Certain Reference Amounts Customarily Consumed; Serving size for Breath Mints; and Technical Amendments: Guidance for Industry Small Entity Compliance Guide [5].[4]Food Sources of Omega-3 Fats Factsheet [6].

Table S3. Correlation matrix of dietary diversity and quality measures.

	Count	Evenness-BI	Evenness-HFBI	Dissimilarity
Count	1	0.565<br>$p < 0.001$	0.167<br>$p < 0.001$	0.209<br>$p < 0.001$
Evenness-BI	0.565<br>$p < 0.001$	1	0.258<br>$p < 0.001$	0.110<br>$p < 0.001$
Evenness-HFBI	0.167<br>$p < 0.001$	0.258<br>$p < 0.001$	1	−0.043<br>$p = 0.049$
Dissimilarity	0.209<br>$p < 0.001$	0.110<br>$p < 0.001$	−0.043<br>$p = 0.049$	1
MAR	0.529<br>$p < 0.001$	0.434<br>$p < 0.001$	0.073<br>$p < 0.001$	0.222<br>$p < 0.001$
DASH	−0.003<br>$p = 0.898$	−0.140<br>$p < 0.001$	0.270<br>$p < 0.001$	−0.348<br>$p < 0.001$

Abbreviations: BI—Berry Index, DASH—Dietary Approaches to Stop Hypertension, HFBI—health value adjusted Berry Index, MAR—Mean Adequacy Ratio.

Table S4. Correlation matrix of food attributes.

	**EPA/DHA**	**Sodium**	**Alcohol**	**Fiber**	**Whole Grains**	**Refined Grains**	**Animal Protein**	**Plant Protein**	**Oils**	**Solid Fats**
**EPA/DHA**	1	0.177	−0.054	−0.262	−0.071	0.015	0.334	−0.099	0.064	0.096
**Sodium**	0.177	1	−0.248	0.089	0.098	0.317	0.341	−0.026	0.300	0.338
**Alcohol**	−0.054	−0.248	1	−0.098	−0.038	−0.098	−0.112	−0.037	−0.118	−0.141
**Fiber**	−0.262	0.089	−0.098	1	0.193	0.019	−0.268	0.096	0.164	−0.076
**Whole Grains**	−0.071	0.098	−0.038	0.193	1	0.159	−0.133	0.134	0.098	−0.082
**Refined Grains**	0.015	0.317	−0.098	0.019	0.159	1	0.305	0.045	0.304	0.289
**Animal Protein**	0.334	0.341	−0.112	−0.268	−0.133	0.305	1	0.004	0.143	0.458
**Plant Protein**	−0.099	−0.026	−0.037	0.096	0.134	0.045	0.004	1	0.265	−0.004
**Oils**	0.064	0.300	−0.118	0.164	0.098	0.304	0.143	0.265	1	0.134
**Solid Fats**	0.096	0.338	−0.141	−0.076	−0.082	0.289	0.458	−0.004	0.134	1

References

1. Salehi-Abargouei, A.; Akbari, F.; Bellissimo, N.; Azadbakht, L. Dietary diversity score and obesity: A systematic review and meta-analysis of observational studies. *Eur. J. Clin. Nutr.* **2016**, 70, 1–9.
2. Dietary Guidelines for Americans 2015–2020 8th Edition. Available online: https://health.gov/dietaryguidelines/2015/guidelines/ (accessed on 5 Mar 2019).
3. Scientific Report of the 2015 Dietary Guidelines Advisory Committee. Available online: https://health.gov/dietaryguidelines/2015-scientific-report/(accessed on 29 April 2019).
4. Food Patterns Equivalents Database 2013–14: Methodology and User Guide. Available online: https://www.ars.usda.gov/ARSUserFiles/80400530/pdf/fped/FPED_1314.pdf (accessed on 29 April 2019).
5. Guidance for Industry: Food Labeling: Serving Sizes of Foods That Can Reasonably Be Consumed At One Eating Occasion; Dual-Column Labeling; Updating, Modifying, and Establishing Certain Reference Amounts Customarily Consumed; Serving Size for Breath Mints. Available online: https://www.fda.gov/regulatory-information/search-fda-guidance-documents/guidance-industry-food-labeling-serving-sizes-foods-can-reasonably-be-consumed-one-eating-occasion (accessed on 29 April 2019).
6. Food Sources of Omega-3 Fats. Available online: https://www.dietitians.ca/getattachment/de95e92c-3fb3-40db-b457-173de89bdc3a/FACTSHEET-Food-Sources-of-Omega-3-Fats.pdf.aspx (accessed on 29 April 2019).
